# Effects of chemical and biological warfare agent decontaminants on trace survival: Impact on fingermarks deposited on paper

**DOI:** 10.1111/1556-4029.70286

**Published:** 2026-02-20

**Authors:** Isabelle Radgen‐Morvant, Natalie Kummer, Christophe Curty, Olivier Delémont

**Affiliations:** ^1^ École des Sciences Criminelles University of Lausanne Lausanne Switzerland; ^2^ Commissariat Forensique Police Neuchâteloise Neuchâtel Switzerland; ^3^ Laboratoire Spiez Federal Office for Civil Protection (FOCP) Spiez Switzerland

**Keywords:** Chemical Biological Radiological and Nuclear (CBRN), decontaminants, indanedione‐zinc, Ninhydrin, oil red O, paper, physical developer, porous surfaces

## Abstract

Chemical and biological events present a challenging environment for forensic scientists to perform their work. This research is the follow‐up of the previous article that investigated the impact of decontaminants on fingermarks deposited on glass. In the present study, the focus shifted to paper, a porous and sensitive substrate where fingermark residues are absorbed and cannot be optically seen, making their detection more difficult. Paper is also highly relevant in forensic casework, as demonstrated by the anthrax letter events. Here the impact of 16 decontaminants on fingermarks deposited on paper and on the subsequent development with Ninhydrin, Indanedione‐zinc, Physical Developer and Oil Red O was evaluated (by visual examination) by four evaluators. The results of the study demonstrated that the outcome of the development technique is influenced by the persistence of fingermarks compounds targeted by the development technique. As expected, the enhancement and detection methods that target amino acids, that is, Ninhydrin and Indanedione‐Zinc had little success when liquid decontaminants were applied to the traces. Although the Physical Developer method revealed more promising results, this technique is generally less reliable compared to others. Therefore, results obtained using this method should be interpreted with greater caution but showed a good chance of success for many decontaminants. Additional tests were conducted using the Oil Red O development technique to evaluate the impact of decontaminants on the lipidic components of fingermarks. Similar to the findings with Physical Developer, the results obtained with Oil Red O showed promising recovery potential and should be further explored.

## 1 INTRODUCTION

In recent years, global security discussions have increasingly focused on digital threats and cybercriminality, driven by rapid technological advancements [1]. While this emphasis on cyber issues is crucial, it can sometimes overshadow the ongoing significance of kinetic threats, including those involving chemical, biological, radiological, and nuclear (CBRN) agents [2]. The successive health crises of Ebola, COVID‐19, and mpox [3, 4] along with ongoing conflicts in Ukraine [5] and in the Levant region [6] have heightened awareness of biological risks but also reignited concerns about potential CBRN incidents and hazardous material exposure.

Although large‐scale bioterrorism remains a relatively low‐probability threat, the risk of targeted attacks or accidental releases involving dangerous substances persists. Incidents such as the anthrax letters of 2001 [7] underscored the need for effective preparedness and the development of forensic techniques capable of responding to such scenarios.

Pursuing research on the potential for fingermark recovery from paper remains valuable in today's context, especially since first responders often encounter threat letters with suspicious material, although, fortunately, many of these turn out to be hoaxes [8, 9].

Such items, along with other paper‐based documents encountered at CBRN incident scenes, can carry critical forensic information. In such scenarios, these documents are often handled by first responders and may be exposed to interventions like fire extinguisher use [10, 11, 12, 13], decontamination, or other emergency procedures, all of which can compromise latent fingermarks. Environmental factors, including temperature, humidity, and general weathering, can further degrade these traces [14, 15, 16, 17, 18].

Understanding how these operational and environmental stresses affect fingermark persistence is crucial, as recovered marks can provide valuable investigative leads and help identify potential perpetrators. Given its role as a common substrate for documents and correspondence, paper is particularly relevant in forensic investigations, as its porous structure can retain fingermark residues over extended periods, supporting long‐term detection and analysis [19]. A better understanding of these effects is essential to develop reliable recovery techniques and demonstrating effective recovery techniques can enhance the forensic support of criminal investigations.

Some decontamination methods have already been studied for their impact on fingermark recovery on paper. Electron beam and gamma irradiation have been explored as decontamination methods for paper, with electron beam negatively affecting fingermarks by modifying their chemistry, resulting in a noticeable decline in both the quality and quantity of ridge details [20]. In contrast, gamma irradiation appears to have no detrimental impact [21, 22]. Decontamination with formaldehyde gas degrades amino acids; however, Physical Developer has been shown to remain effective for fingermark enhancement on paper exposed to it [23]. Other decontamination methods, such as ozone, dry fogging, chlorine dioxide, MODEC MDF‐500, and Bioxy S, have also been shown to hinder fingermark detection techniques targeting amino acids, such as Indanedione‐Zinc (IND‐Zn) and 1,8‐Diazafluoren‐9‐one (DFO) [22]. Vaporized hydrogen peroxide (VHP) is considered a good alternative, as it had minimal effect on fingermark recovery with DFO and IND‐Zn [22]. In corrosive environments, such as those involving exposure to alkaline or acidic solutions, Physical Developer has proven to be the most effective technique for recovering fingermarks on porous surfaces, while techniques like Oil Red O and Iodine fuming were less successful, likely due to their sole reliance on the fatty components of fingermarks, which are susceptible to degradation in such conditions [24].

Building on a previous investigation into the effects of decontamination on fingermarks on glass [25], this follow‐up study investigates the impact of decontamination on fingermark detection on paper while broadening its scope to include biological decontamination methods. This study encompasses the 10 decontaminants evaluated in the previous work, along with 6 additional decontamination agents.

The study is divided into two parts. The first part studies the performance of Ninhydrin (NIN), Indanedione‐Zinc (IND‐Zn), and Physical Developer (PD) on decontaminated traces from nine volunteers, representing a range of donor variability, categorized as good, medium, and poor. The second part focuses on a subset of three donors from the original group, to further investigate on the performance of Physical Developer and Oil Red O (ORO). Development methods targeting different components of the fingermarks (i.e., amino acids or hydrophobic fraction) were considered for this study.

Ninhydrin and Indanedione‐Zinc are commonly used techniques that react with amino acids, while Physical Developer and Oil Red O target the hydrophobic fractions of the fingermark [19, 26, 27]. Since amino acids are soluble in polar solvents, methods like Ninhydrin and Indanedione‐Zinc tend to be less effective on wetted substrates [19, 28]. In contrast, Physical Developer is better suited for developing fingermarks on wet porous surfaces, although it requires significant resources and does not always yield consistent results [17, 19, 26, 29]. ORO, which targets the lipidic portion of fingermarks, is also a suitable technique for wetted fingermarks [17, 19, 27, 29] but can be less effective when organic solvents or cleaning agents have been used, as these can remove the reaction sites [24]. Further details on each method are provided in the respective sections.

## 2 MATERIALS AND METHODS

### 2.1 Fingermark deposition

The study was divided into two parts to assess the impact of the 16 decontaminants on fingermarks and fingermarks development techniques.

For the first part, focusing on NIN, IND‐Zn and PD, fingermarks were deposited by nine volunteers (five women and four men aged between 20 and 30 years). In the second part, which examined PD and ORO, three volunteers (one woman and two men aged between 23 and 30 years) participated. In both parts, each participant deposited three fingermarks (index finger, middle finger, and ring finger) on pre‐cut sheets of white office paper (Canon Black Label Zero, 80 g/m^2^). This resulted in 27 fingermarks per decontaminant and per development technique in part 1, and 9 fingermarks in part 2 (Part 1: 27 fingermarks × 16 decontaminants × 3 development techniques = 1296 fingermarks (or 2592 half‐fingermarks) and Part 2: 9 fingermarks × 16 decontaminants × 2 development techniques = 288 fingermarks (or 576 half‐fingermarks)).

Donors were instructed to wash their hands 30 min before deposition and to gently rub the middle three fingertips of each hand together to ‘homogenize’ the secretion residues prior to deposition. The fingermarks were then stored in a cupboard under atmospheric conditions (20–25°C and ~30%–50% relative humidity) for 2 days before decontamination.

### 2.2 Decontamination

A total of 16 decontaminants targeting chemical and biological agents was selected for testing (Table 1). Ten of these were common to the previous study on glass [25]: water, soapy water, isopropanol, Alldecont, BX24, RSDL®, GDS 2000, GD‐6, FastAct®, and CH‐Powder. For the present study, two additional chemical agent decontaminants were included (Commercial Bleach and Wasa®‐Soft & Clorina®), as well as a neutralizing agent called SkinNeutrAll® bleach. In addition, three decontaminants targeting biological agents (Wofasteril, Vaprox, and Virkon®S) were tested.

**TABLE 1:** Decontaminants used and their properties and application parameters.

Main target	Main chemical mechanism	Decontaminants (manufacturer/supplier)	Actives substances	Application	Exposure time
Chemical agents	Physical Removal	Water^a^ (from the tap)		Immersion	5 min
		Soap Water^a^ ~ 0.1% (v/v) (Tap water with “Handy” detergent^b^)		Immersion	5 min
		Isopropanol^a^ (Sigma‐Aldrich > 99.8%)		Immersion	5 min
		SkinNeutrAll®^a^ (Ilma Biochem)	Ascorbic acid	Spray	5 min
	Absorption Adsorption Oxidation	CH‐Powder (Swiss Armed Forces Command Support Organisation)	Chlorinated lime, Magnesium oxide	Powder	90 s
		FastAct® (Enware)	Magnesium oxide, Titanium oxide	Powder	90 s
	Nucleophilic substitution	GDS2000 (Kärcher)	Aminoethanol	Spray	10 min
		GD6 (OWR)	2‐Aminoethanol, Diethylenetriamine	Spray	15 min
		RSDL® (Emergent Bio)	2,3‐Butadiene monoxime	recovered	2 min
	Oxidation	Alldecont (OWR)	Sodium hypochlorite	Spray	2 min
		BX24 ~ 10% (w/v) (Cristianini)	Dichloroisocyanurate	Immersion	15 min
		Commercial Bleach (Potz)	Sodium hypochlorite	Immersion	5 min
		Wasa®‐Soft & Clorina® ~ 2.5% (v/v & w/v) (Lysoform)	Tosylchloramide	Immersion	2 min
Biological agents	Oxidation	Virkon®S ~ 1% (w/v) (Lanxess)	Pentapotassium bis(peroxymonosulfate)‐bis(sulfate), Benzenesulfonic acid	Immersion	10 min
		Vaprox diluted ~ 10% (v/v) (Steris)	Hydrogen peroxide	Immersion	15 min
		Wofasteril ~ 2% (v/v) (Kesla)	Peracetic acid	Spray	60 min

^a^ Investigated as removal method, but with hydrolysis capabilities.

^b^ “Handy” is a Swiss dish soap detergent brand manufacturer by Migros.

Each fingermark was divided in half, with one half subjected to decontamination. The papers with the half‐fingermarks were placed in glass trays with the marks facing upward. Some decontaminants (i.e., AllDecont, GSD2000, GD‐6, Wofasteril, SkinNeutrAll®) were sprayed onto the papers until these were fully saturated. For other liquid decontaminants (i.e., water, soap water, isopropanol, bleach, Vaprox, Virkon®S, Wasa®‐Soft & Clorina®, BX24), the papers were immersed in 2–3 cm of solution. Powdered decontaminants (FastAct® and CH‐Powder) were applied until the papers were completely covered. For RSDL®, the gel was squeezed from the sponges and spread over the papers to ensure full coverage.

The half‐fingermarks were exposed to the decontaminants for periods ranging from 1 to 60 min, following the manufacturer's instructions (Table 1). After decontamination, the papers were removed, laid out on a laboratory bench, and left to dry overnight.

### 2.3 Development of fingermarks

After decontaminating one half of each fingermark, both halves were subsequently developed. In the first part of the study, Ninhydrin, Indanedione‐Zinc, and Physical Developer were applied. In the second part, Physical Developer was used again, along with Oil Red O.

The impact of each decontamination procedure was assessed by comparing the development results of the two halves of each fingermark, the one decontaminated prior to development, and the other directly developed with the same technique (serving as reference/control). The side (left or right) treated with the decontaminant was randomly assigned for each fingermark.

#### 2.3.1 Ninhydrin

The ninhydrin solution was prepared following the protocol described by Champod et al. (2004) [27]. Formulation details are presented in Table 2. Before each application, the solution was tested using a SEMA NIN/DFO/IND test strip. After immersion, fingermarks were placed in a heat and humidity chamber at 80°C and 65% humidity for 10 to 20 min. Developed fingermarks were observed and photographed under white light with a Canon EOS 6D camera equipped with a Canon Compact‐Macro EF 50 mm lens combined with a Life Size Converter EF.

**TABLE 2:** Formulation used to prepare the different fingermark development solutions.

Ninhydrin (1 L) [30]	1,2‐indanedione/zinc (1 L) [31]	Physical developer [1 L] [32]	Oil red O (1 L) [19, 33]
Working solution: 4 g ninhydrin (BVDA) 20 mL methanol (Sigma‐Aldrich, 99.8%) 70 mL ethyl acetate (Sigma‐Aldrich, 99.5%) 10 mL acetic acid (Sigma‐Aldrich) 900 mL petroleum ether (Honeywell)	Working solution: 0.25 g 1,2‐indanedione (BVDA) 100 mL ethyl acetate (Sigma‐Aldrich, 99.5%) 100 mL methanol (Sigma‐Aldrich, ≥99.8%) 10 mL acetic acid (Sigma‐Aldrich, ≥ 99%) 800 mL petroleum ether (Honeywell) 20 mL ZnCl_2_ solution ZnCl_2_ solution: 0.2 g ZnCl_2_ (Sigma‐Aldrich) 100 mL ethanol (Sigma‐Aldrich, ~ 96%)	Working solution: 900 mL PD‐Redox solution 50 mL PD‐DGME solution 50 mL PD‐AgNO_3_ solution PD‐Redox solution: 900 mL RO/DI water 30 g Iron (III) nitrate nonahydrate (Sigma‐Aldrich ≥98%) 80 g Ammonium iron(II) sulphate hexahydrate (Sigma‐Aldrich ≥98.0%) 20 g citric acid monohydrate (Sigma‐Aldrich) PD‐DGME detergent solution: 1 L RO/DI water 1.5 g n‐dodecylamine acetate (Pfaltz & Bauer) 1.25 g DGME (Decaethyleneglycol monododecyl ether) (Sigma‐Aldrich) PD‐AgNO_3_ solution: 50 mL RO/DI water 10 g silver nitrate (Sigma‐Aldrich ≥99.0%) Maleic acid solution: 50 g maleic acid (Sigma‐Aldrich ≥99%) 2 L RO/DI water	Working solution: 1.54 g Oil Red O (Sigma‐Aldrich) 770 mL methanol (Sigma‐Aldrich, 99.8%) 230 mL NaOH solution NaOH solution: 9.2 g NaOH (Merck) 230 mL water

#### 2.3.2 Indanedione‐zinc

The indanedione‐Zinc solution was prepared according to the formulation described in Bonnaz et al. [31]. Formulation details are presented in Table 2. Before each application, the solution was tested using a SEMA NIN/DFO/IND test strip. The fingermarks were immersed for a few seconds in the IND‐Zn solution, air‐dried, and processed for 10 seconds in a heat press at 165°C. Developed fingermarks were observed and photographed using a 532 nm laser (TracER Compact laser ‐ Coherent, USA) and a 550 nm high‐pass orange filter attached to a Canon EOS 6D camera equipped with a Canon Compact‐Macro EF 50 mm and Life Size Converter EF.

#### 2.3.3 Physical developer

The Physical Developer solutions were prepared and applied following the protocol by Bleay et al. [32]. Formulation details are presented in Table 2. Before each application, the solution was tested using a tera‐sodium EDTA spot test as recommended by Houlgrave and Ramotowski [34].

For the first part of the study, the half‐fingermarks (27 traces per decontaminant) were immersed in the working solution for 20 min. In Part 2 (9 traces per decontaminant), immersion times were extended and adjusted based on control results of the donors. Specifically, traces from two donors were immersed for 30 min, while traces from the third donor, which exhibited light development, were immersed for 40 min.

After development, fingermarks were air‐dried and then observed and photographed using a Crime‐lite AUTO (Foster+Freeman) with integrated lighting under white light, as well as under a LED 850 nm infrared (IR) lighting with an IR 780 nm long pass filter.

#### 2.3.4 Oil red O

The ORO solution was prepared following the recipe by Beaudoin and Champod et al. [19, 33]. Formulation details are presented in Table 2. Before each application, the solution was tested using a control fingermark.

The immersion times varied based on individual donor characteristics observed in control traces: fingermarks from the first donor were immersed for 30 min, while those from the second and third donors were immersed for 90 min. Notably, both the decontaminated and corresponding control half‐fingermarks were immersed for the same duration to ensure consistency in the development process.

After immersion, fingermarks were rinsed with deionized water and left to air dry for 24 h before being photographed under white light with a Canon EOS 6D camera equipped with a Canon Compact‐Macro EF 50 mm and a Life Size Converter EF.

In the second part of the study, immersion times were personalized to better assess and optimize fingermark recovery for each donor. Tailoring immersion times to the unique characteristics of each donor's secretion composition allowed for enhanced development efficacy. Implications of this individualized approach will be further elaborated in the Results and Discussion section.

### 2.4 Quality assessment of fingermarks

Both halves of each fingermark were photographed side‐by‐side under the same lighting conditions mentioned above for each development technique. Each half‐fingermark's quality was independently evaluated by four evaluators based on the captured images, using the absolute grading system outlined by the Center for Applied Science and Technology (CAST) [35, 36, 37], as detailed in Table 3.

**TABLE 3:** Description of the absolute quality grading scale (CAST Scale) used for the half‐fingermarks [35, 36, 37].

Score	Definition
0	No evidence of fingermark
1	Some evidence of fingermark
2	Less than 1/3 clear ridge detail
3	Between 1/3 and 2/3 clear ridge detail
4	Over 2/3 clear ridge detail

The relative evaluation system developed by the University of Canberra (UC) (ranging from −2 to 2) was used to directly compare the development of the decontaminated half of each fingermark to its reference corresponding half [35, 37]. This system is presented in Table 4. Four evaluators independently assessed the quality by comparing the two halves. To minimize bias, the images were presented in a randomized order with no indication or labeling identifying the treatment conditions.

**TABLE 4:** Description of the relative quality grading scale (called UC‐00) used to evaluate the impact of the decontaminants. Adapted from [35, 37].

Score	Definition
+2	Half‐fingermark developed by method A after decontamination exhibits far greater ridge detail and/or contrast than the corresponding half‐fingermark developed by method A without decontamination
+1	Half‐fingermark developed by method A after decontamination exhibits slightly greater ridge detail and/or contrast than the corresponding half‐fingermark method A without decontamination
0	No significant difference between the corresponding half‐fingermarks
‐1	Half‐fingermark developed by method A without decontamination exhibits slightly greater ridge detail and/or contrast than the corresponding half‐fingermark developed by method A with decontamination
‐2	Half‐fingermark developed by method A without decontamination exhibits far greater ridge detail and/or contrast than the corresponding half‐fingermark developed by method A with decontamination
00	No trace mark is visible on neither half‐fingermark developed with method A

### 2.5 Data treatment

Each half‐fingermark was assigned four quality scores, one from each evaluator. Instead of averaging these scores, it was decided to retain and use all four values for subsequent analyses, to preserve the full range of evaluator assessments.

**FIGURE 1:**
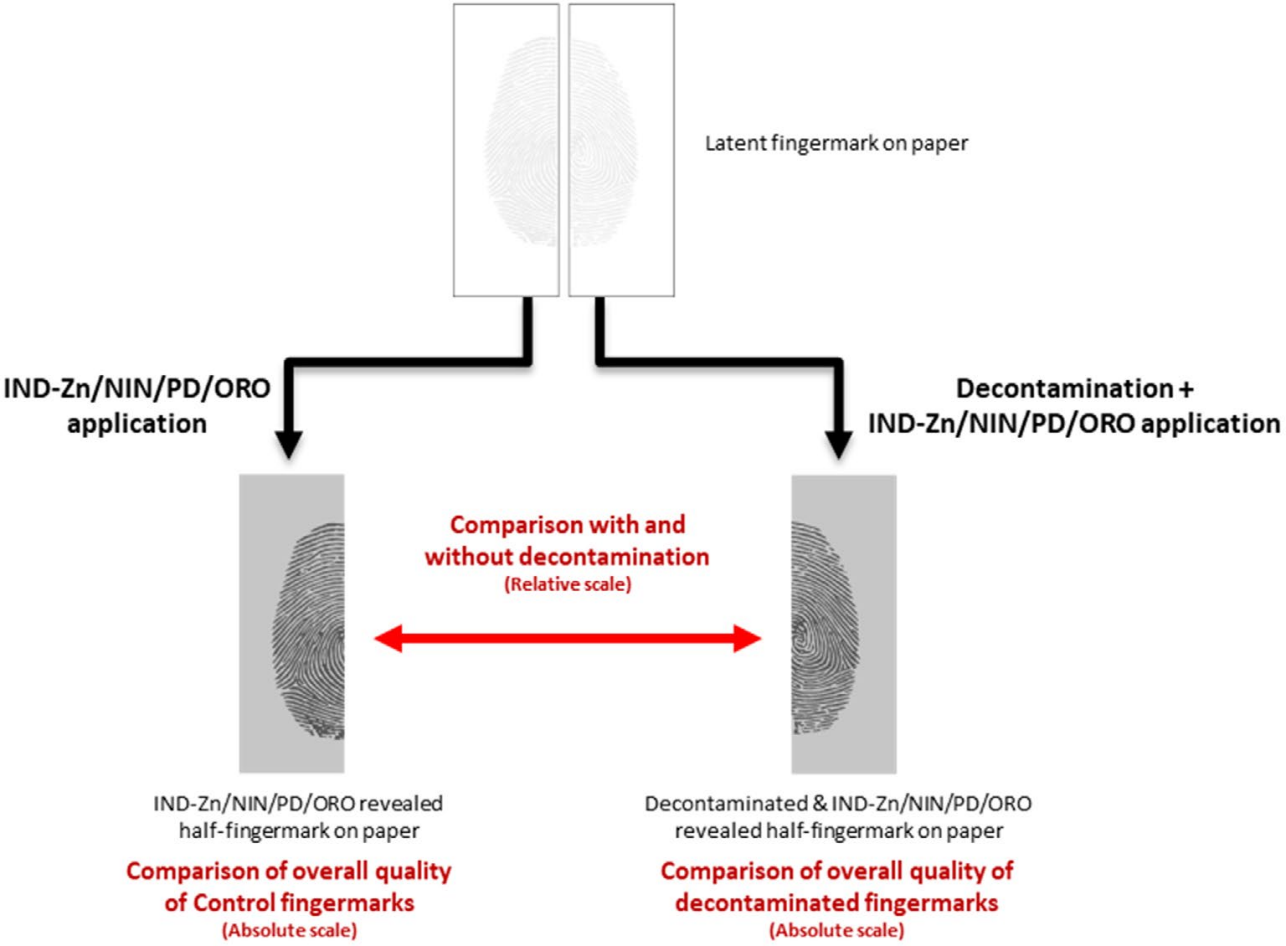
Methodological sequence for evaluating the impact of decontaminants on fingermarks on porous substrates, indicating where the CAST absolute scoring scale was applied and how the relative UC‐00 scale was used to compare half‐fingermarks.

For each pair of half‐fingermarks, the decontaminated and developed half was compared to the control half (only developed) using a relative grading system. The resulting scores indicated which development techniques were more suitable for fingermark recovery for each of the selected decontaminants, thereby assessing the impact of decontaminants on fingermarks deposited on paper. The data treatment workflow is summarized in Figure 1.

## 3 RESULTS AND DISCUSSION

### 3.1 Quality of fingermarks

It is important to note that the quality assessment of the fingermarks in this study was solely based on the images captured through optical examination after development. Unlike fingermarks deposited on glass (previously published study [25]), it was not possible to determine the quality of the traces on paper prior to their reaction with the development technique. To better reflect real‐case scenarios, where traces often vary in quality and may be partial, a natural deposition process was used, producing fingermarks of varying quality. To ensure comparability, fingermarks were observed and photographed under standardized lighting and photographic conditions.

The results of the control fingermarks from each donor showed variability in the quality, reflecting individual differences in fingermark deposition typical of natural fingermarks. It should be noted that the scores were attributed to half‐fingermarks, which likely resulted in lower overall scores compared to what would be expected for full fingermarks. Indanedione‐Zinc demonstrated the highest sensitivity, followed by Ninhydrin and the Physical Developer.

### 3.2 Performance of Ninhydrin to develop fingermarks following decontamination

**FIGURE 2:**
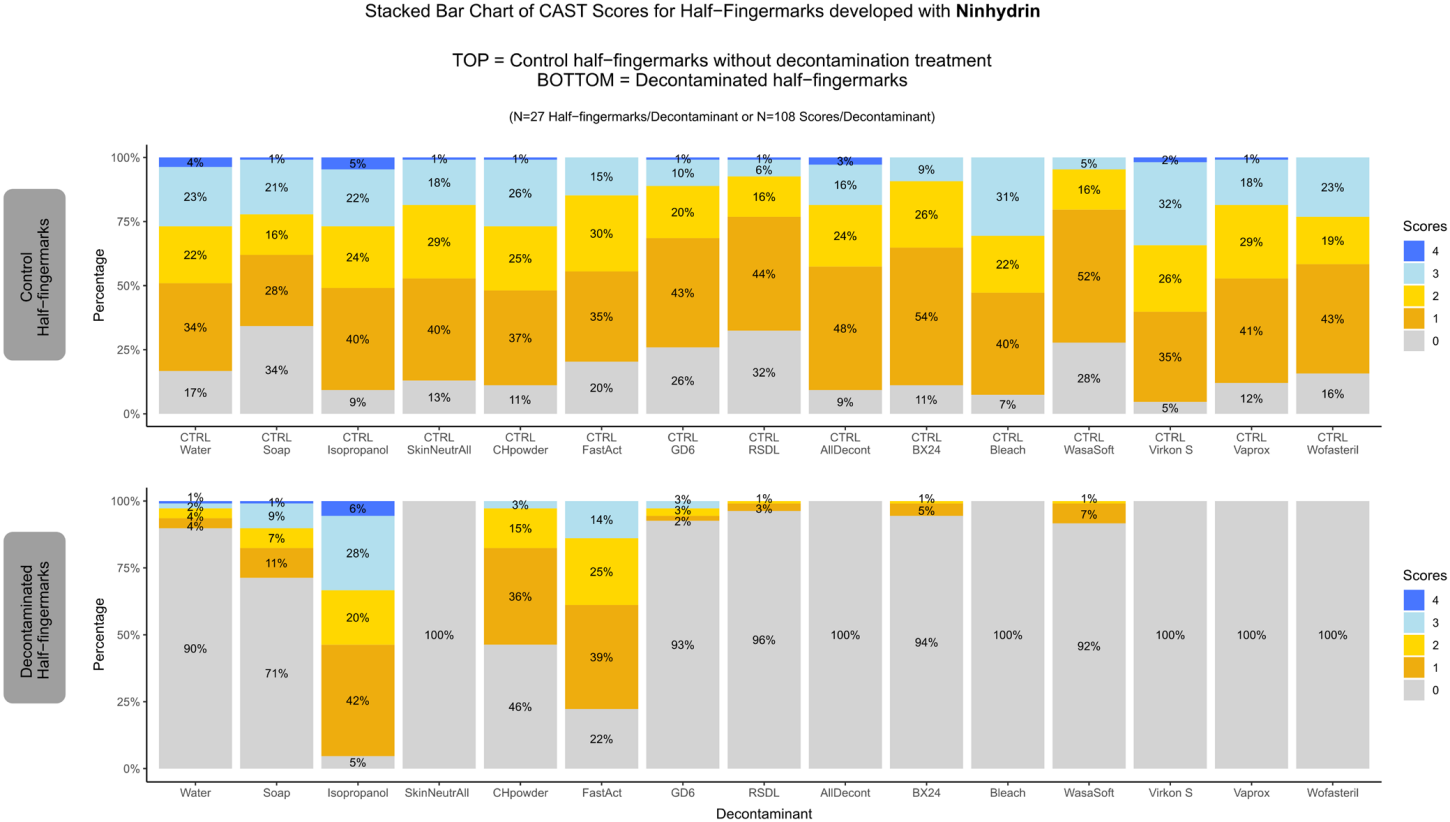
An example of a fingermark developed with ninhydrin is shown for each decontaminant. The left side displays the control half‐fingermark developed with ninhydrin without decontamination, and the right side shows the corresponding decontaminated half‐fingermark developed with ninhydrin. Where necessary, images were mirrored to maintain a consistent left–right alignment of control and decontaminated impressions.

The half‐fingermarks composing the control group developed with ninhydrin exhibited overall homogeneity in quality. The variability observed in the scores reflects the natural variation typically encountered in real‐life fingermark samples, and the occurrence of approximately 5%–30% of marks scoring 0 is consistent with the known sensitivity of the ninhydrin process (Figure 2).

**FIGURE 3:**
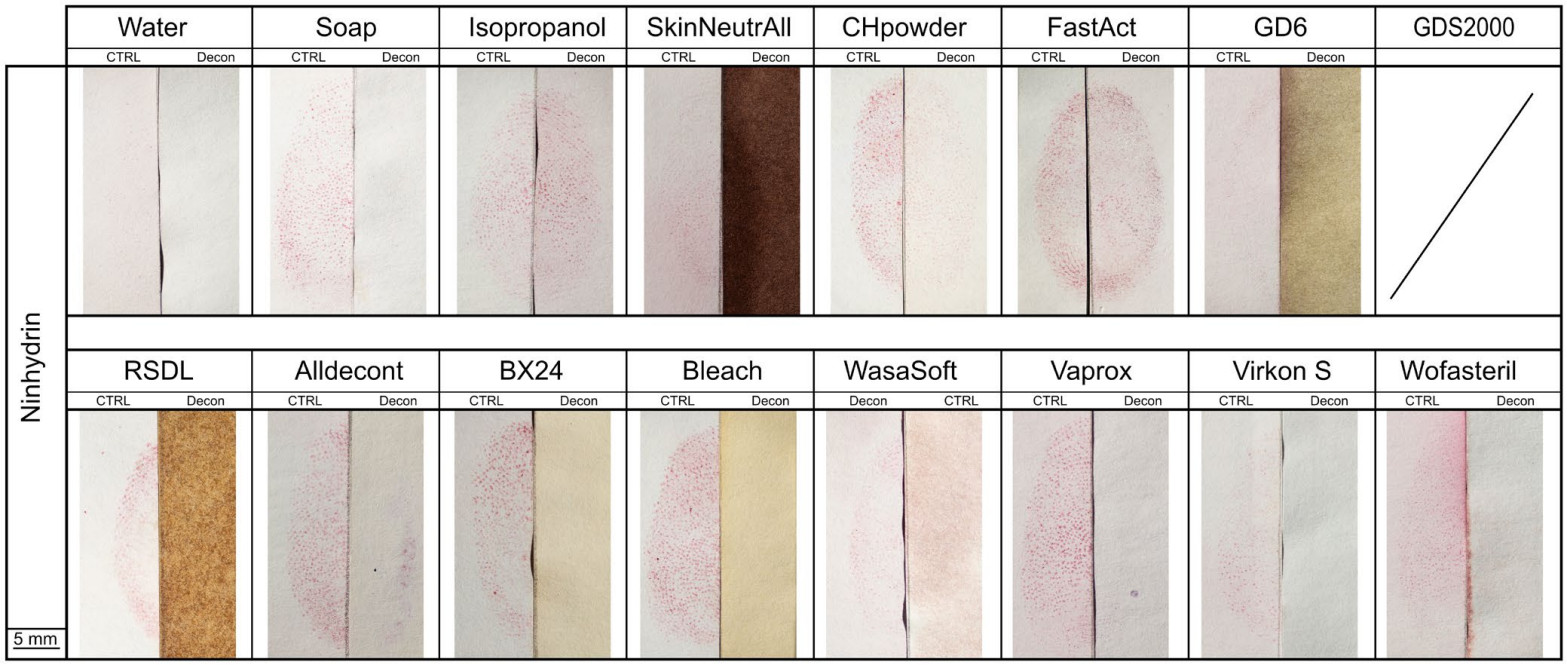
CAST Results for ninhydrin developed half‐fingermarks. On the top: Control half‐fingermark. On the bottom: Half‐fingermark decontaminated and developed with ninhydrin.

Initial observations revealed that Ninhydrin reacted strongly with certain decontaminants, causing visible changes to the paper substrates. In particular, papers treated with SkinNeutrAll®, GDS2000, GD‐6 and RSDL® darkened noticeably following the application of Ninhydrin and exhibited further darkening after exposure in the heat and humidity chamber (Figure 3). GD6 and GDS200 have amino‐functional components in the formulation which can be expected to react with ninhydrin, leading to the characteristic purple‐blue coloration of the Ruhemann's purple [28]. The use of GDS2000 resulted in an unusually strong reaction during the Ninhydrin development in the heat and humidity chamber, causing the entire paper to turn dark purple. This effect was so pronounced that even non‐decontaminated control paper subsequently turned purple. To restore normal chamber function, extensive cleaning with water, soap, and acetone, followed by several hours of blank operation, was necessary. Consequently, no usable result could be obtained for GDS2000 decontaminated fingermarks on paper.

Both BX24 and bleach, and Alldecont to a lesser extent, caused paper discoloration. These background changes not only hindered the developed fingermarks but also raised concerns about potential interactions between the development methods and decontaminants in general.

Both BX24 and bleach, and Alldecont to a lesser extent, caused noticeable paper discoloration. These background changes hindered fingermark development and can be explained by the interaction of chlorine‐based oxidizing agents with the paper substrate. Paper is primarily composed of cellulose and residual lignin, both of which are susceptible to oxidative reactions [38]. Oxidation of paper constituents by OCl^−^ can convert hydroxyl groups into carbonyl and carboxyl functionalities and generate conjugated oxidation products, altering the chemical structure of the fibers and leading to yellowing or browning of the paper [38, 39, 40], as observed in this study.

When examining the decontaminated and subsequently developed half‐fingermarks with Ninhydrin, only three decontaminants, namely isopropanol, FastAct®, and CH‐Powder, resulted in good fingermark recovery, with over 50% of the half‐fingermarks being detected for these three decontaminants (score >1) (Figure 2).

As alcohols are commonly incorporated into amino acid–reactive reagent formulations, they are not expected to chemically modify or degrade amino acids present in latent fingermark residues. Previous research has demonstrated that ethanol exposure does not inhibit the performance of 1,2‐indanedione, indicating that alcohols exert minimal impact on amino acid‐targeting detection techniques [41]. Consistent with these findings, ninhydrin was observed to effectively develop fingermarks following exposure to isopropanol, with little loss in quality.

Although the two powders CH‐Powder and FastAct®, which mechanism is based on absorption adsorption oxidation, had a greater effect on Ninhydrin development than isopropanol, they still allowed for good fingermark recovery. It can be hypothesized that these powders have only a limited ability to permeate the paper structure, thus protecting the amino acids trapped within and allowing for subsequent detection. Nevertheless, CH‐Powder proved to be more detrimental to fingermark recovery on paper than FastAct®, a conclusion supported by the results from Indandione‐Zinc. This difference is clear in the relative grading scale comparing decontaminated and developed half‐fingermarks, to their respective controls where 63% of FastAct®‐treated half‐fingermarks were rated as equal in quality to controls, while only 20% CH‐Powder‐treated half‐fingermarks received the same rating (Figure S1).

All other decontaminants, which were liquid and included several water‐based options, did not support the development of fingermarks with Ninhydrin following decontamination. Consistent with previous studies, these decontaminants likely interact with amino acids, with water‐based solutions solubilizing them and removing target sites for Ninhydrin [19, 28].

### 3.3 Performance of indanedione‐zinc to develop fingermarks following decontamination

**FIGURE 4:**
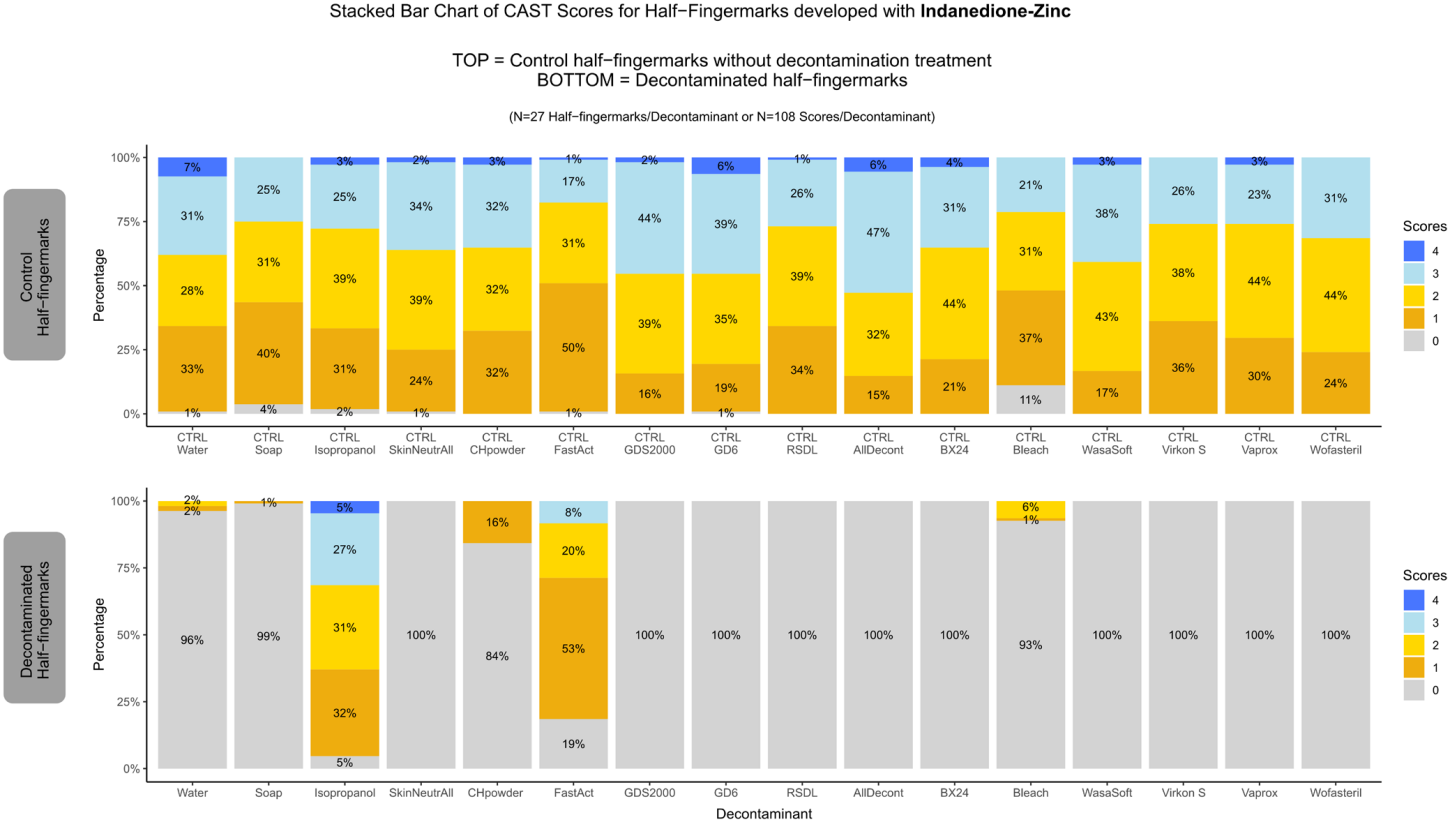
CAST Results for Indanedione‐Zinc developed half‐fingermarks. On the top: Control half‐fingermark. On the bottom: Half‐fingermark decontaminated and developed with indanedione‐zinc.

In terms of fingermark development with Indanedione‐Zinc, the overall scores for the Control half‐fingermarks consistently scored higher than those developed with Ninhydrin. Notably, there were almost no half‐fingermarks rated with a score of 0 in the control group developed with Indanedione‐Zinc (Figure 4).

No strong background coloration was observed after application of 1,2‐indanedione‐Zn. However, paper that had been treated with chlorine‐containing decontaminants (bleach, CH‐Powder, Wasa®‐Soft & Clorina®, BX24, AllDecont®) exhibited a more pronounced yellowish hue, whereas paper decontaminated with SkinNeutrAll® developed a brown tint. As previously discussed for Ninhydrin, these discolorations are consistent with reactions between the paper substrate and active chlorine‐releasing oxidants from the decontaminants, which can modify lignocellulosic components and lead to yellowing or browning of the paper [38, 39, 40].

Regarding the decontaminated half‐fingermarks, results were similar to those observed with Ninhydrin, which aligns with expectations since both techniques target the same compounds. However, fingermarks treated with CH‐Powder showed a notable difference: While some half‐fingermarks could still be developed with ninhydrin at a quality comparable to controls, Indanedione–Zinc failed to develop them effectively. This highlights the complex chemistry involved in successful detection, and the intricate interplay among fingermark components, decontaminants, and development techniques. Such findings underscore the need for further research to enhance our understanding of the underlying chemistry.

Isopropanol and FastAct® were the only decontaminants that maintained good recovery rates, yielding fingermark detections with indanedione‐zinc that were comparable in quality to the untreated controls (Figure S2).

### 3.4 Performance of physical developer to develop fingermarks following decontamination

#### 3.4.1 Part 1

In the first set of fingermarks developed with the Physical Developer (Part 1 of the study), it was noted that many half‐fingermarks failed to develop, even within the control group. A significant number of these control half‐fingermarks, between 44% and 93%, received a score of 0, indicating no visible traces (Figures S3 and S4). The poor development of the control fingermarks complicated the interpretation of trends and the evaluation of decontaminant effects on fingermarks development. It also prevented the determination of whether the Physical Developer could serve as an alternative to Ninhydrin and Indanedione‐Zinc.

Beyond the scoring results, several visual observations were made during the application of the Physical Developer to this first set of fingermarks. GD6 and GDS2000 had a notably detrimental impact on the paper substrate when combined with the PD procedure. In the pre‐washing bath with maleic acid, papers decontaminated with these two agents began to dissolve, which caused significant decomposition and prevented any subsequent fingermark development.

GD6 and GDS2000 are highly alkaline, nucleophilic, non‐aqueous decontamination formulations containing strong bases such as potassium or sodium hydroxide and reactive alkoxides [42, 43, 44]. Maleic acid, a dicarboxylic acid, is used in pre‐washes to neutralize alkaline fillers such as calcium carbonate [26]. When residues of these alkaline decontaminants remain within a cellulose‐based substrate and are then exposed to a maleic acid wash, local pH gradients can develop, driving base‐catalyzed peeling reactions in areas rich in decontaminant [45, 46] and acid‐catalyzed hydrolysis of glycosidic bonds where the acid reacts with fillers and fibers [46, 47, 48]. Together, these processes markedly accelerate fiber weakening and degradation and, in the present study, provide a plausible explanation for the severe disintegration of the paper matrix that was observed.

**FIGURE 5:**
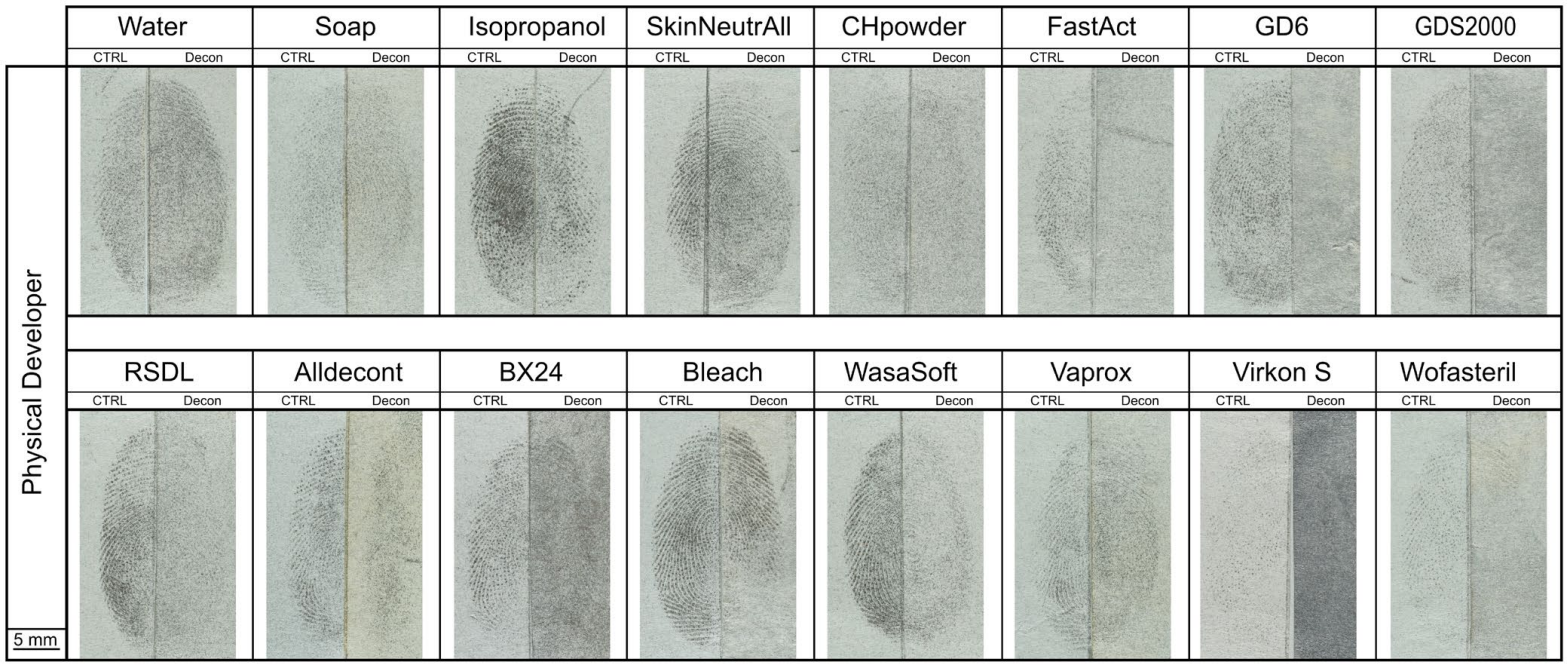
An example of a fingermark developed with physical developer is shown for each decontaminant. The left side displays the control half‐fingermark developed with physical developer without decontamination, and the right side shows the corresponding decontaminated half‐fingermark developed with physical developer. Where necessary, images were mirrored to maintain a consistent left–right alignment of control and decontaminated impressions.

Papers decontaminated with Virkon®S turned completely black within minutes of immersion in the silver‐containing bath, necessitating earlier removal from the development bath (Figure 5). Blackening of Virkon®S‐treated papers in PD suggests silver deposition from non‐selective reduction of silver ions to metallic silver. This increased background coloration greatly reduced contrast, making fingermark detection difficult.

For paper treated with bleach, marbling patterns were observed, and the substrate became brittle, although less severely than with GD6 and GDS2000. Similar marbling was noted with BX24 and Alldecont®. Paper decontaminated with SkinNeutrAll® developed air bubbles between its layers during the maleic acid pre‐washing bath, producing darker spots at the end of the development process.

To gather more comprehensive data and confirm these initial observations, experiments with the Physical Developer were repeated with three donors, those who provided the highest number of detectable fingermarks after decontamination and could therefore be considered as “good donors”. This aimed to enhance understanding of the potential of the Physical Developer in this context.

#### 3.4.2 Part 2

As mentioned, only three donors were included in this second part, and the development time in the silver bath was extended to ensure effective development. The results confirmed the observations made during the first part, as illustrated in Figure 5.

The control half‐fingermarks yielded good results, allowing for clear comparison with those that had been decontaminated prior to development with the Physical Developer.

**FIGURE 6:**
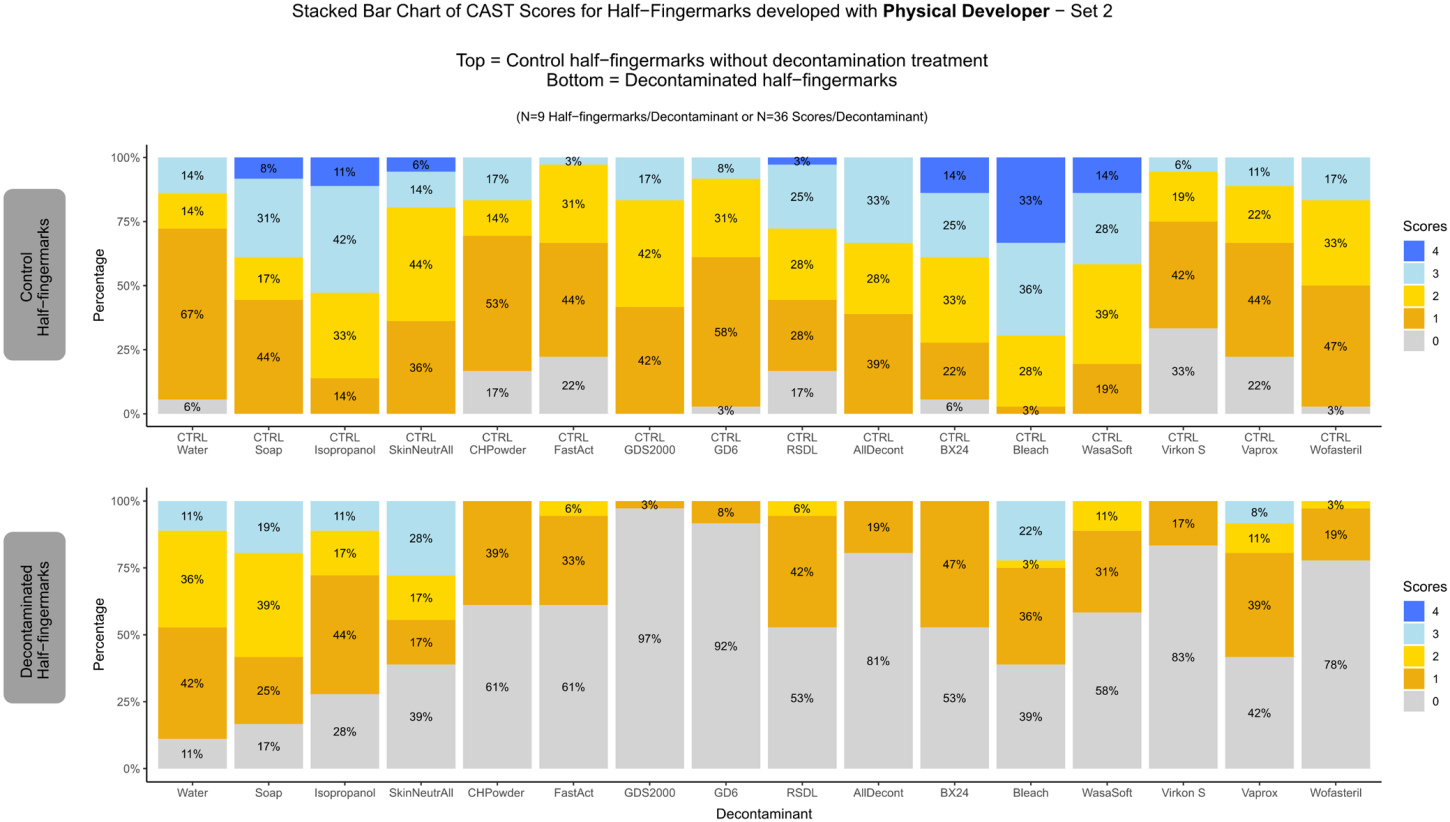
CAST Results for Physical Developer developed half‐fingermarks. On the top: Control half‐fingermark. On the bottom: Half‐fingermark decontaminated and developed with Physical Developer.

The decontamination procedures based on removal methods (i.e., water, soap, isopropanol, and SkinNeutral®) had only a minor impact on fingerprint recovery (Figure 6), demonstrating that the Physical Developer, which targeted the non‐hydrosoluble fraction of fingermarks, can effectively develop wetted fingermarks, as previously reported in the literature [19, 49, 50].

Overall, it appears that development after decontamination with the Physical Developer proved more successful than the two amino acid‐targeting techniques tested (NIN and IND‐ZN). However, it should be noted that GDS2000 and GD6 are incompatible with the Physical Developer, as the maleic acid bath led to the dissolution of paper decontaminated with these agents. RSDL® exhibited less detrimental effects, allowing detection of 47% of the traces after decontamination (Figure 6).

For decontaminants based on oxidation process applied by immersion (BX24, bleach and Wasa®‐Soft & Clorina®), between 42% and 61% of half‐fingermarks were recovered, whereas the spray‐applied AllDecont® yielded only 19% recovery. Physical Developer generally yielded poor results in developing fingermarks after the application of decontaminants targeting biological agents (Virkon®S, Vaprox, and Wofasteril). However, whereas no fingermarks were recovered with Ninhydrin or Indanedione‐Zinc, Physical Developer was able to partially recover fingermarks following Vaprox treatment, with 19% of traces achieving a score of ≥2, thereby performing better than the amino acid‐targeting methods.

When considering the relative scale, it becomes apparent that, with the exception of the physical removal treatments and the powder‐based formulations, the decontaminants overall reduced the effectiveness of fingermark development with the physical developer (Figure S5).

### 3.5 Performance of Oil Red O to develop fingermarks following decontamination

Finally, the effect of the decontaminants on Oil Red O development was assessed using fingermarks from the same donors selected for the second test with the Physical Developer.

**FIGURE 7:**
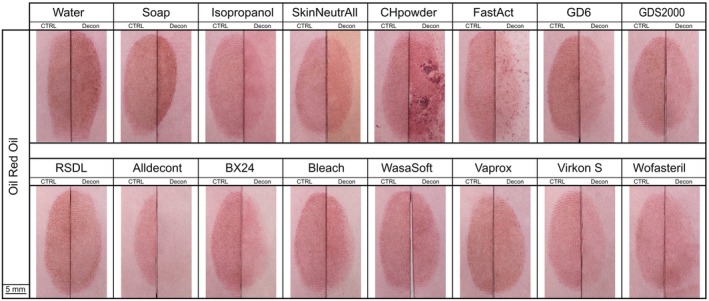
An example of a fingermark developed with Oil Red O is shown for each decontaminant. The left side displays the control half‐fingermark developed with Oil Red O without decontamination, and the right side shows the corresponding decontaminated half‐fingermark developed with Oil Red O. Where necessary, images were mirrored to maintain a consistent left–right alignment of control and decontaminated impressions.

**FIGURE 8:**
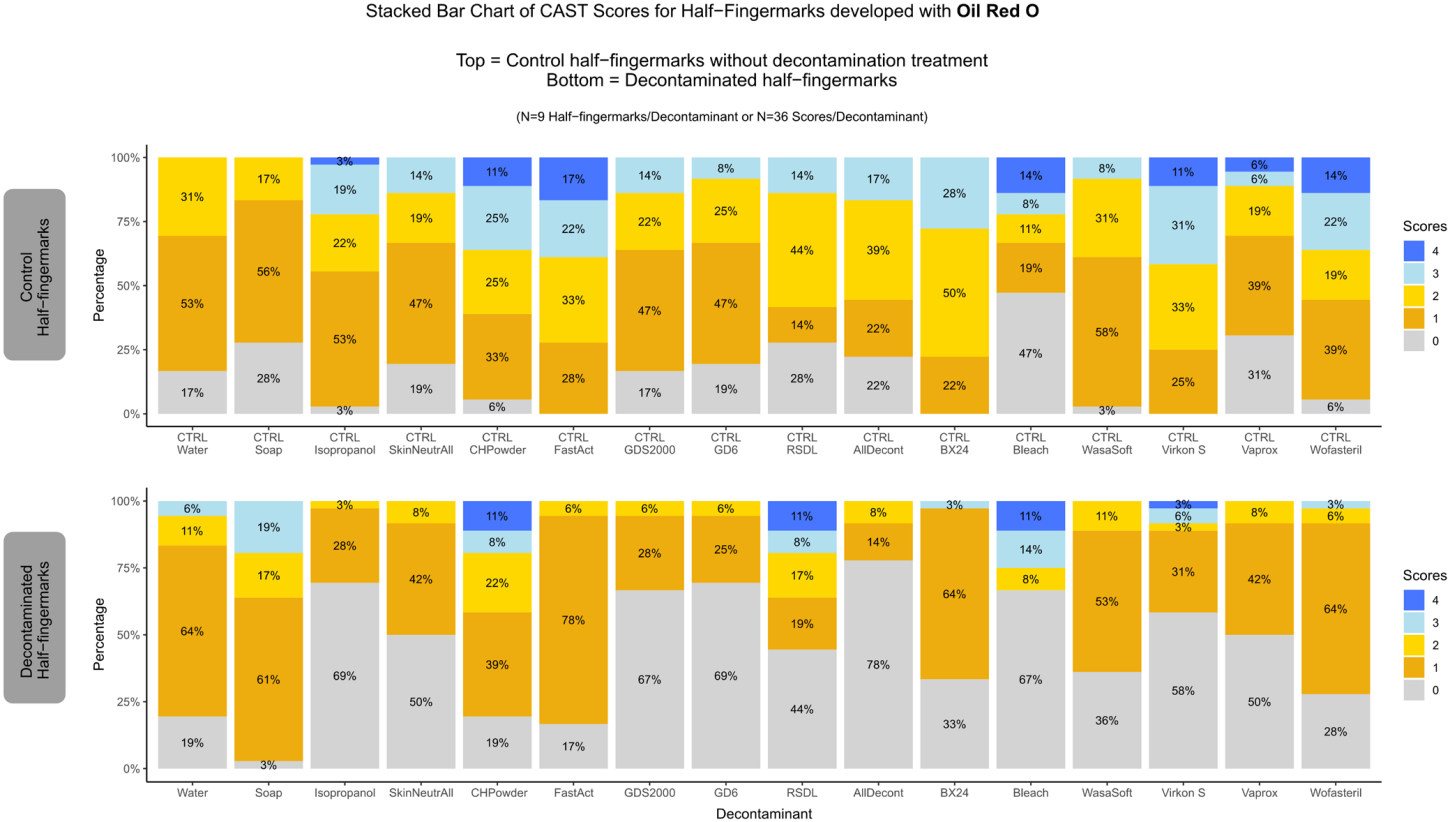
CAST Results for Oil Red O developed half‐fingermarks. On the top: Control half‐fingermark. On the bottom: Half‐fingermark decontaminated and developed with Oil Red O.

Among the tested decontaminants and enhancement methods, Oil Red O (ORO) yielded promising results for fingermark recovery of decontaminated fingermarks (Figure 7). Most decontaminants, including water, soap, CH‐Powder, FastAct®, RSDL®, BX24, Wasa®‐Soft & Clorina®, Vaprox, Wofasteril, allowed detection of 50% or more of the half‐fingermarks after decontamination. Water and soap did not negatively impact recovery, and even slightly improved the quality of some fingermarks (Figure 8 and Figure S6). RSDL® and CH‐Powder also showed promising results, with half of the decontaminated traces exhibiting equal or higher quality compared to controls (Figure S6). After FastAct® decontamination, more than 50% of the half‐fingermarks were developed (Figure 8), but these were generally of lower quality. Notably, 78% of FastAct®‐treated half‐fingerprints developed with ORO scored only 1, and evaluation of control half‐fingermarks for FastAct® were of noticeably higher quality.

Among chemical decontaminants based on nucleophilic substitution (i.e., GDS2000, GD6, and RSDL), the two containing a primary alcohol (GDS2000, GD6) negatively affected fingermark development. Similarly, isopropanol clearly hindered trace recovery with ORO. Such results may be explained by the dissolution of sebaceous compounds, the target compounds of ORO, in alcohol [51].

GDS2000, GD6, and Alldecont® reduced the clarity and contrast of the developed fingermarks (Figure 7), resulting in generally poor outcomes with ORO.

Within the group of oxidation‐based chemical decontaminants (Alldecont®, BX24, commercial bleach, and Wasa®‐Soft & Clorina®), bleach demonstrated the best results. Fingermarks developed with ORO following bleach decontamination were comparable in quality to control half‐fingermarks. In contrast, Alldecont®, despite having the same active compound as bleach (sodium hypochlorite), led to poor development performance.

Similar to observations with the Physical Developer, Oil Red O provided promising recovery rates after treatment with decontaminants targeting biological agents (i.e., Virkon®S, Vaprox, and Wofasteril). Vaprox and Wofasteril, in particular, allowed respectively 39 and 36% of the fingermarks to maintain equal quality with or without decontamination (Figure S6).

## 4 DISCUSSION

Unlike fingermarks on glass, those on paper are often latent and cannot be observed by optical means, necessitating specialized development techniques for their revelation [19]. In this context, it is essential to investigate the compatibility between decontaminants and development techniques.

For Oil Red O development experiments and the second set of fingermarks developed with the Physical Developer, immersion times were tailored individually for each donor. While this approach does not reflect real‐case scenarios, where such information is not available, it was a deliberate choice in this study to maximize fingermark development and specifically assess the impact of the selected decontaminant. The focus was on studying the decontaminant's effect rather than assessing the overall performance of the development techniques across various donors. To achieve this, the optimal immersion time was first determined using the control half‐fingermark and then applied identically to the decontaminated one. This allowed a direct assessment of the decontaminant's effect on the development outcomes. It is acknowledged that, in operational casework, the inability to determine optimal development times a priori may influence fingermark enhancement success.

Overall, the Physical Developer emerged as the most effective technique for enhancing fingermarks decontaminated by physical removal methods such as water, soap water, isopropanol, or SkinNeutrAll®. Fingermark enhancement with ORO performed efficiently after decontamination with water and soap water, while Ninhydrin and Indanedione‐Zinc were found to be better suited for fingermarks decontaminated with isopropanol.

When fingermarks were decontaminated using powders (i.e., CH‐Powder and FastAct®), effective development was achieved with Oil Red O and the amino acid‐targeting techniques (Indanedione‐Zinc and Ninhydrin).

Among the nucleophilic substitution‐based decontaminants (i.e., GDS2000, GD6, and RSDL®), GDS2000 and GD6 showed poor compatibility with paper substrates, causing damages resulting in ineffective fingermark recovery across all tested techniques. Oil Red O, however, still allowed around 30% of the half‐fingermarks to be visible after development, but with most traces showing a lower quality compared to controls.

Fingermarks decontaminated by oxidation‐based agents such as Alldecont®, BX24, commercial bleach, or Wasa®‐Soft & Clorina® were not developed using techniques targeting amino acids (Ninhydrin and Indanedione‐Zinc). However, partial recovery was achieved using methods targeting hydrophobic compounds (Physical Developer) or the lipidic fraction (Oil Red O) of the fingermarks.

For fingermarks exposed to decontaminants targeting biological agents (i.e., Virkon®S, Vaprox and Wofasteril), both the Physical Developer and Oil Red O provided promising results. The Physical Developer was effective following Vaprox decontamination, while Oil Red O demonstrated strong potential in fingermark development after both Vaprox and Wofasteril. Virkon®S was found to be incompatible with the Physical Developer, causing a rapid darkening of the paper substrate.

Chlorine‐based decontaminants notably affected the paper substrates, causing discoloration, and marbling across all development techniques. This not only affected the appearance and the structure of the paper but also compromised the integrity of the fingermarks themselves, making their detection more challenging following such decontamination. And overall, we observed that the decontamination procedures have a negative impact on the recovery of the fingermarks deposited on paper in all our study cases.

Although the present study examined a broad range of decontaminants, other experimental variables were intentionally constrained. Specifically, the fingermarks were only aged for 2 days before decontamination, deposited exclusively on one type of paper substrate, and limited to the first depletion in each series. These limitations mean that aging effects, substrate variability, and depletion sensitivity were not fully explored in this phase.

The current approach was therefore designed as a first exploratory step, primarily to determine whether fingermarks could still be recovered after exposure to a wide variety of decontaminants and to identify the most promising development techniques for each category.

A subsequent phase of research could serve as a valuable next step. Building on the current findings, this stage could focus on a smaller, more targeted selection of decontaminants while systematically incorporating additional variables such as fingermark aging, depletion series, and different paper substrates. It could also further investigate the impact of various parameters related to the application of the decontaminants. As highlighted by Frick et al., the method of application and the condition of the substrate can strongly influence outcomes [17]; for fragile or aged exhibits, certain sequential treatments may not be feasible without causing damage.

It may also be worthwhile to repeat the experiments using complementary sequences, such that techniques targeting amino acids are followed by those aimed at the hydrophobic fraction of fingermarks. This sequential approach could reveal whether combining methods enhances overall fingermark recovery and under which conditions this improvement is most effective.

Finally, it should be recognized that an additional layer of complexity may arise in reality, as the application of a decontaminant assumes the presence of a contaminating agent, which itself brings its own chemical reactivity, an aspect that has not yet been considered in this study.

Considering these aspects in future work will strengthen the interpretation of results and support a more comprehensive understanding of the interplay between contamination, decontamination, substrate properties, and fingermark composition.

## 5 CONCLUSION

This study evaluated the impact of various decontamination methods on the development and detection of fingermarks on paper, reaffirming the complex interactions that can occur between decontaminants and fingermark development techniques. While some outcomes aligned with previously known findings, such as the sensitivity of amino acid‐targeting techniques to substrate wetting and the suitability of PD and ORO for use on such substrates, the study also revealed new insights relevant for forensic field applications in CB events. Notably, FastAct® and isopropanol did not affect fingermark development with Ninhydrin and Indanedione‐Zinc. Conversely, GDS2000 and GD6 hindered both fingermark detection and the integrity of the paper substrate, indicating that these decontaminants should be avoided on paper substrates when subsequent forensic examination is envisioned.

Both Physical Developer and, especially, Oil Red O yielded promising results, demonstrating that fingermark recovery on paper post‐decontamination is possible. In particular, Oil Red O allowed the recovery of high‐quality fingermarks even after treatment with decontaminants such as RSDL® and bleach, which otherwise led to poor outcomes with Ninhydrin, Indanedione‐Zinc, and Physical Developer.

Further research is warranted to explore potential interactions in more complex scenarios, including the presence of contamination agents, is recommended. Overall, this study supports the use of Oil Red O as the preferred development technique for fingermarks on decontaminated paper to maximize successful recovery, except in cases following isopropanol or FastAct® decontamination, where ninhydrin is recommended.

## CONFLICT OF INTEREST STATEMENT

All authors declare that they have no conflicts of interest.

## ETHICS STATEMENT

The study was approved by the institutional ethical review board of the faculty of law, criminal science, and public administration (CER‐FDCA) of the University of Lausanne, Switzerland (Number: E_FDCA_042022_00001, issued on 08.06.2022) and informed consent was obtained from the donors.

## Supporting information


Figure S1–Figure S6.

## Data Availability

The data that support the findings of this study are available on request from the corresponding author. The data are not publicly available due to privacy or ethical restrictions.
