# Supplementary material for: Effects of chemical and biological warfare agent decontaminants on trace survival: Impact on fingermarks deposited on paper
Source: J Forensic Sci. 2026 Feb 20;71(3):1405–19. doi: 10.1111/1556-4029.70286 (PMC13139828; doi:10.1111/1556-4029.70286)
Supplement: Supplementary file 1 — Figure S1–Figure S6. [file JFO-71-1405-s001.docx]

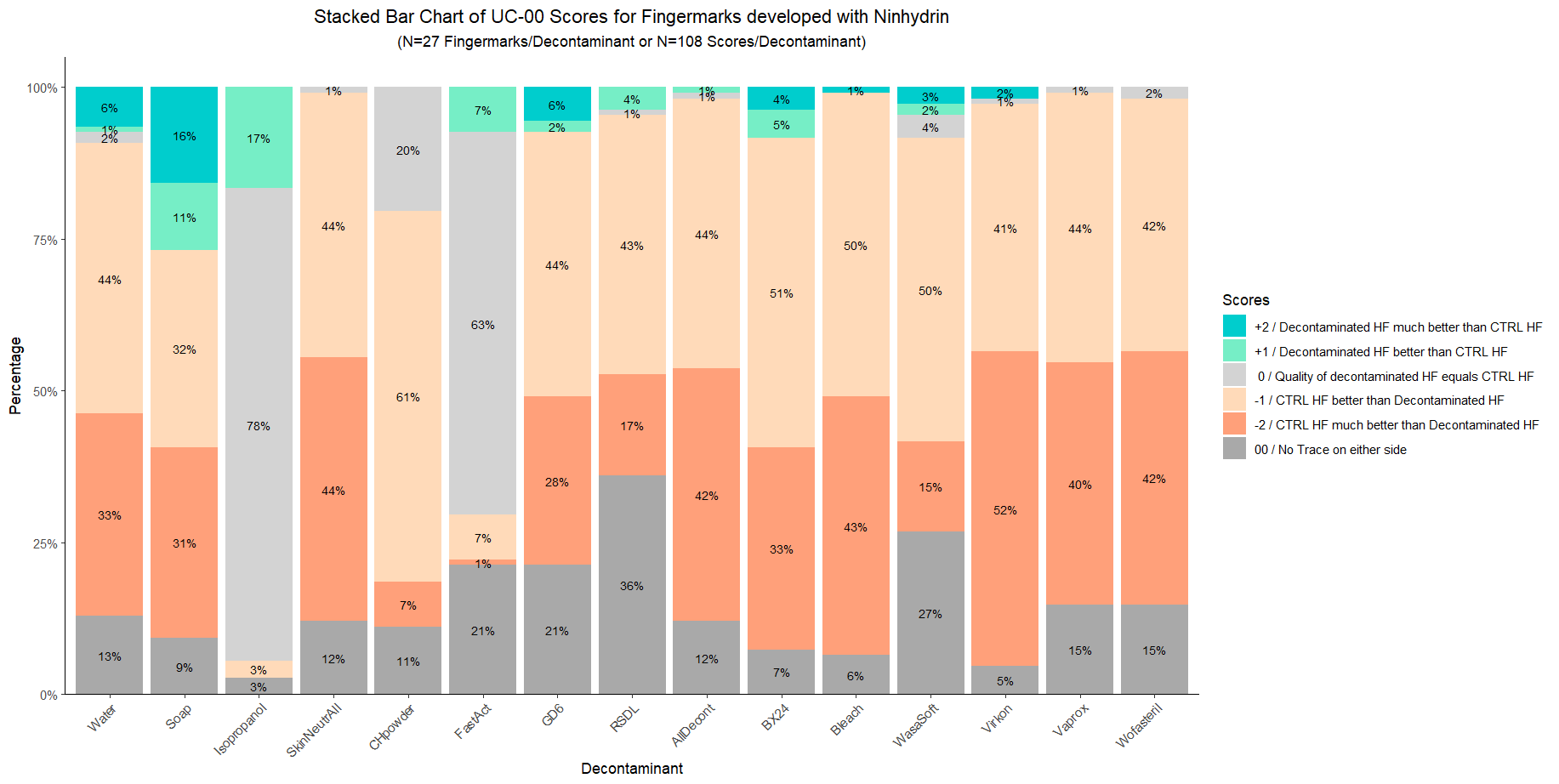


FIGURE S1 UC-00 results for Ninhydrin developed fingermarks. Comparison between control half-fingermarks and decontaminated and ninhydrin developed half-fingermarks.


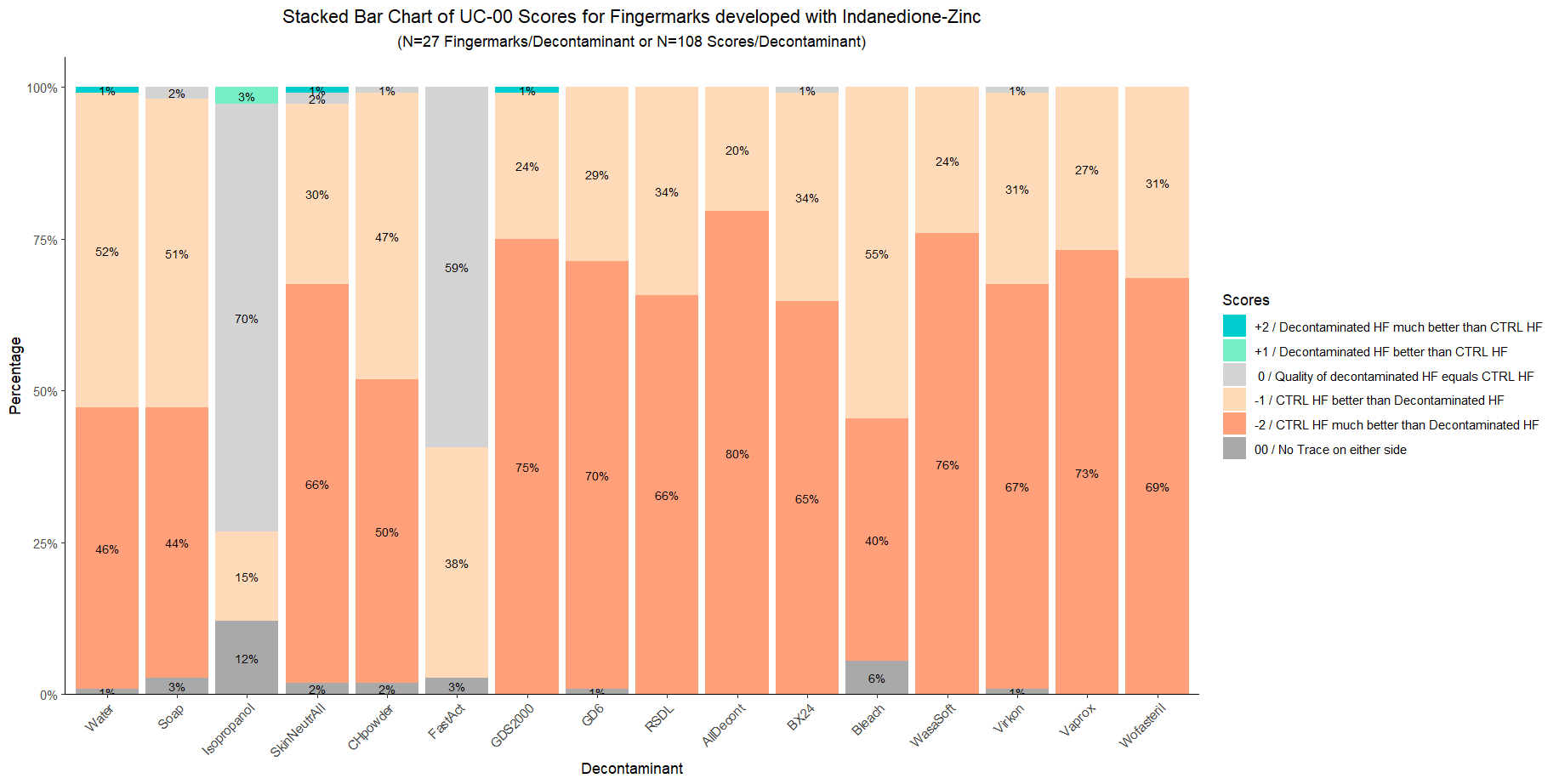


FIGURE S2 UC-00 results for Indanedione-Zinc developed fingermarks. Comparison between control half-fingermarks and decontaminated and indanedione-zinc developed half-fingermarks.


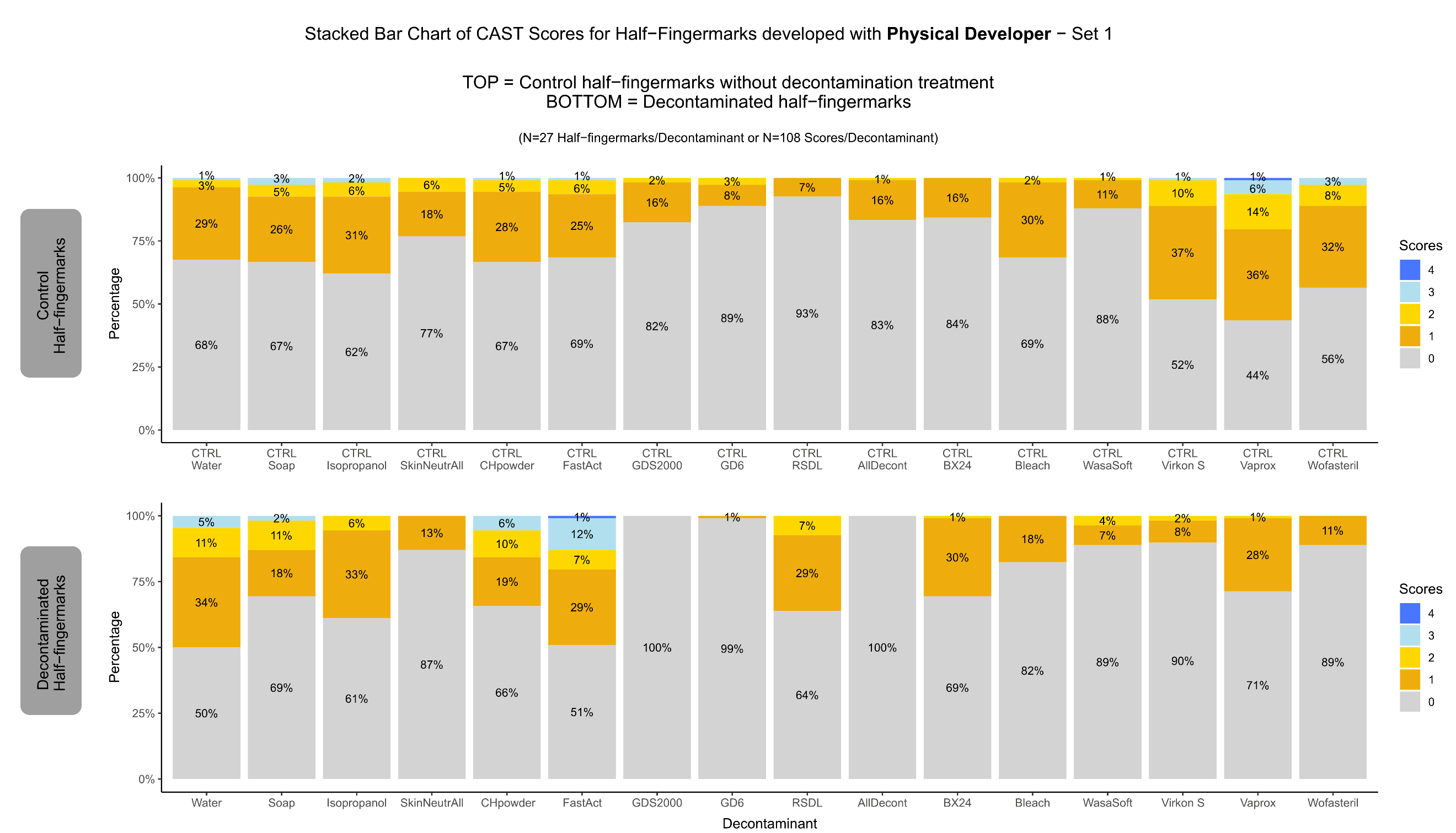


FIGURE S3 CAST Results for Physical Developer developed half-fingermarks - set 1. On the top: control half-fingermark. On the bottom: half-fingermark decontaminated and developed with physical developer.


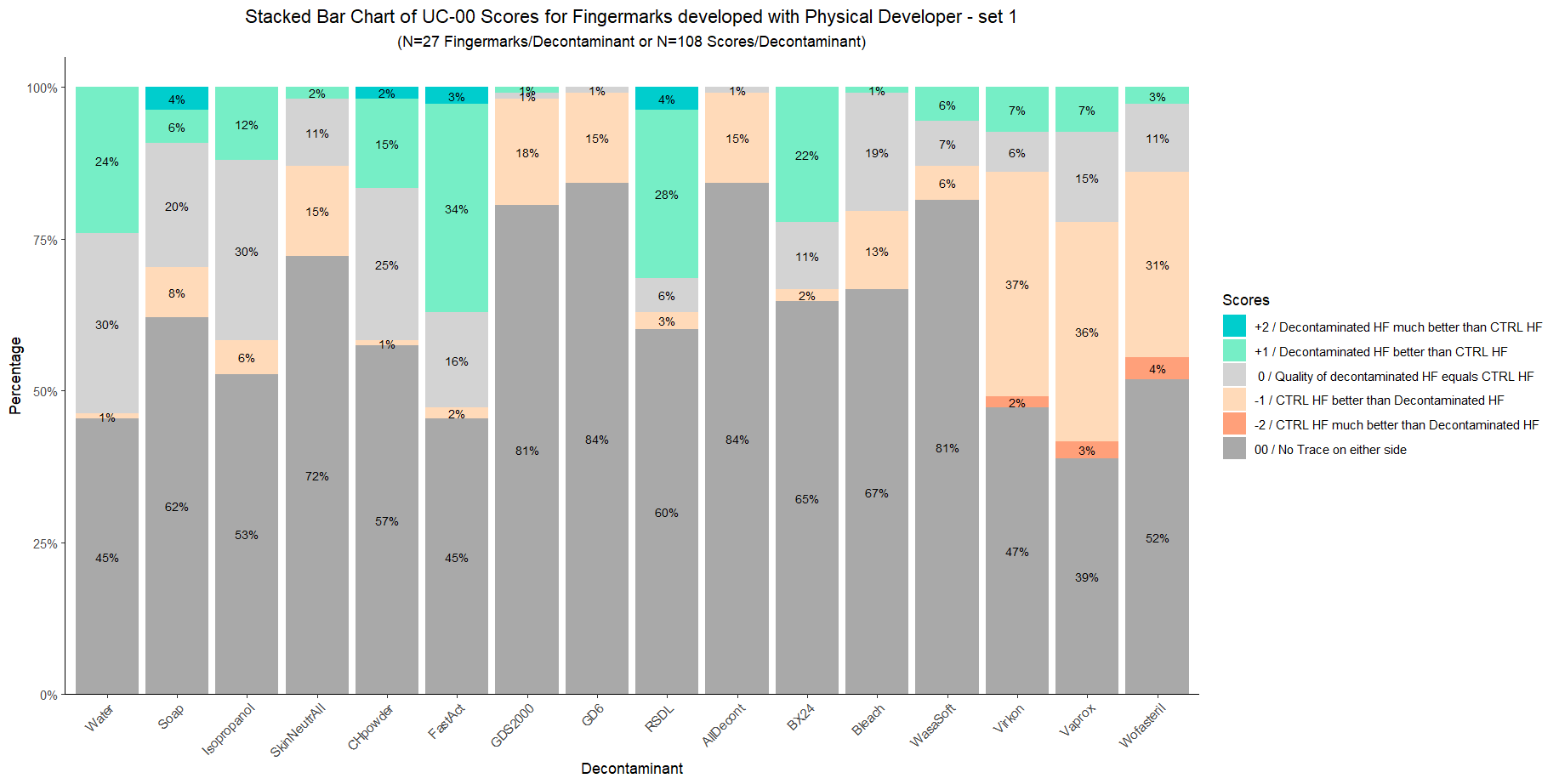


FIGURE S4 UC-00 results for Physical Developer developed fingermarks - set 1. Comparison between control half-fingermarks and decontaminated and Physical Developer developed half-fingermarks.

*
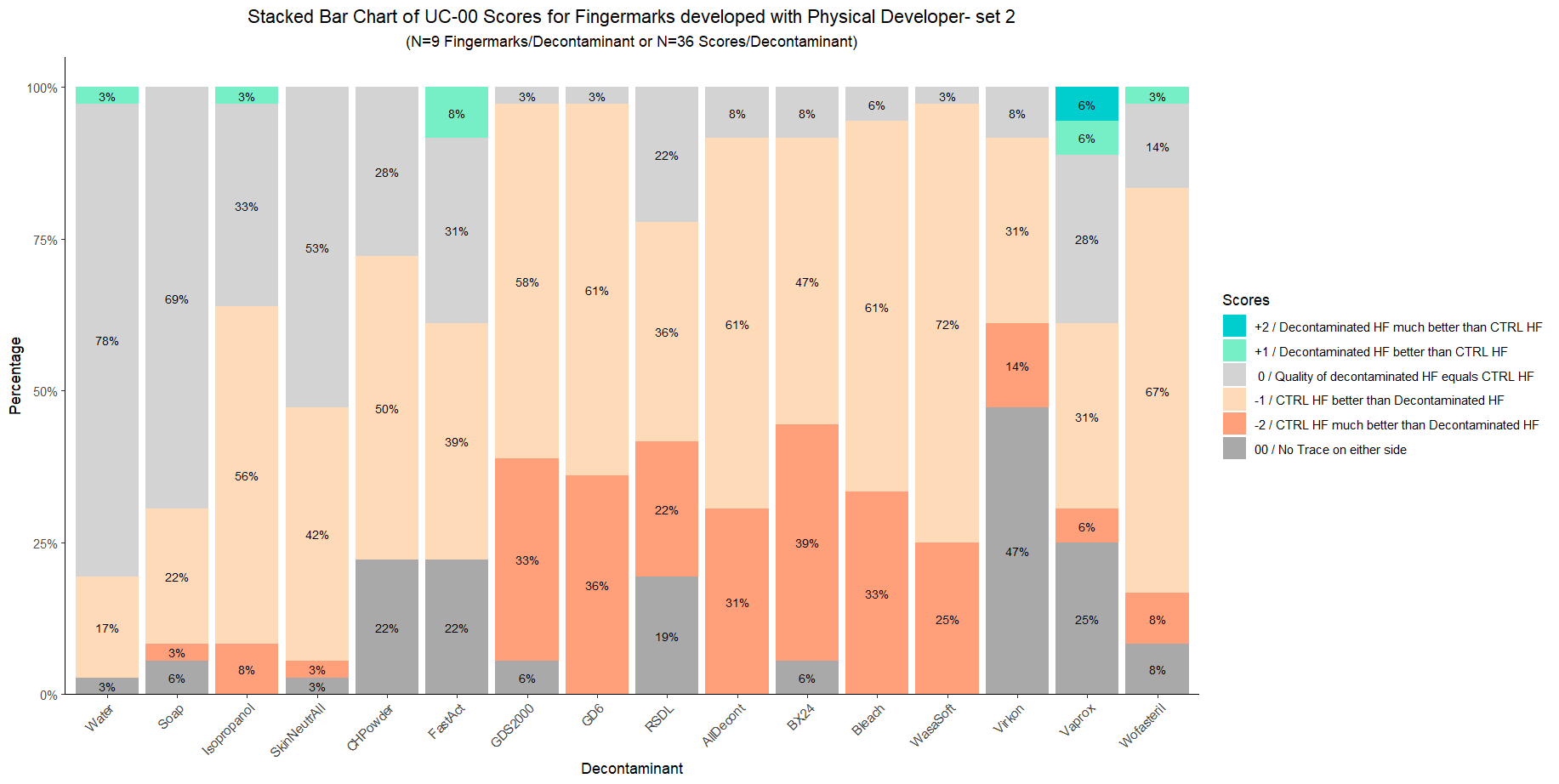
*

FIGURE S5 UC-00 results for Physical Developer developed fingermarks - set 2. On the top: control half-fingermark. On the bottom: half-fingermark decontaminated and developed with physical developer.

*
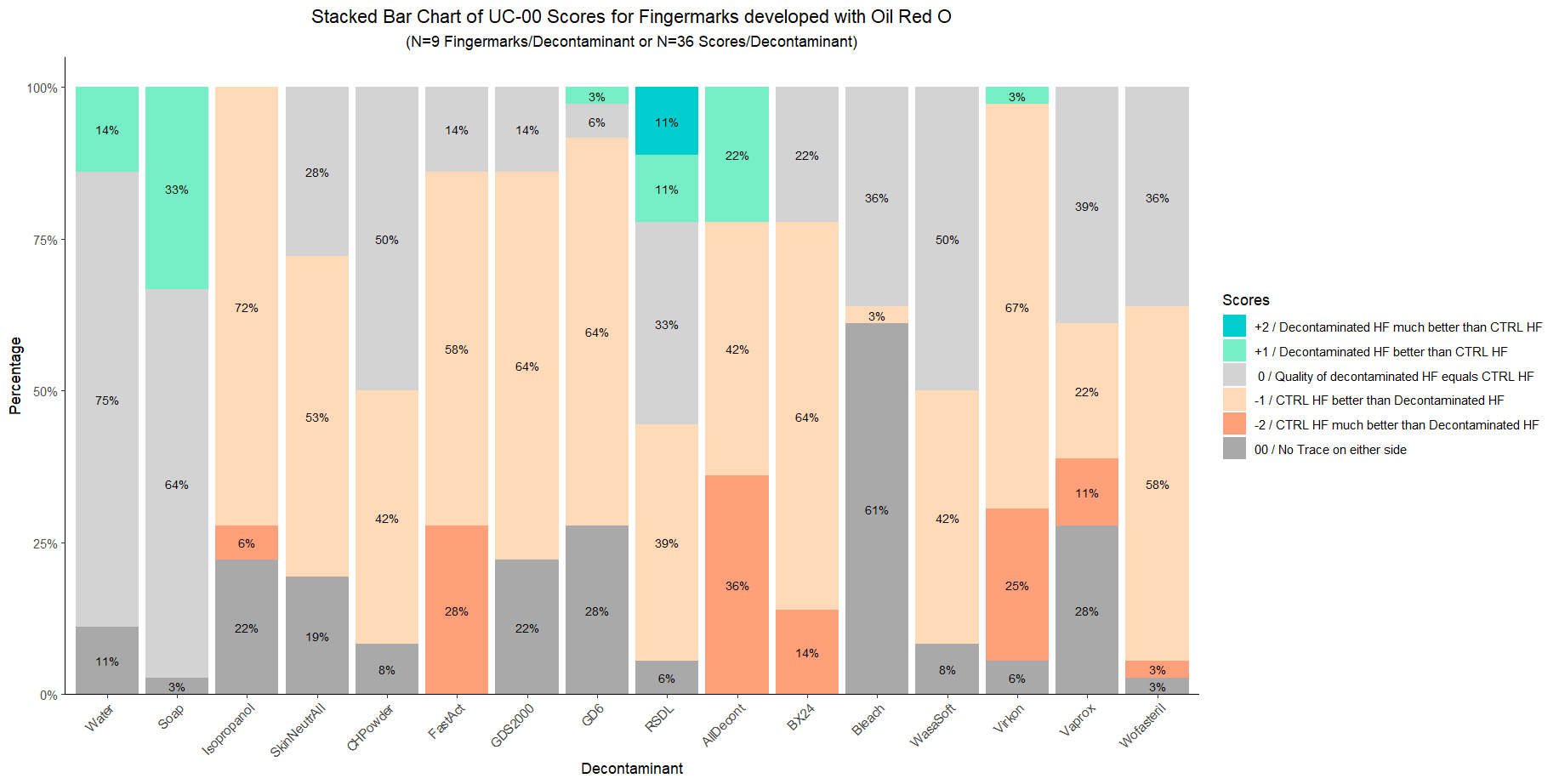
*

FIGURE S6 UC-00 results for Oil Red O developed fingermarks. Comparison between control half-fingermarks and decontaminated and Oil red O developed half-fingermarks.
